# Effects of Riboflavin Collagen Crosslinker on Dentin Adhesive Bonding Efficiency: A Systematic Review and Meta-Analysis

**DOI:** 10.3390/ma16041701

**Published:** 2023-02-17

**Authors:** Sumaiya Zabin Eusufzai, Aparna Barman, Nafij Bin Jamayet, Wan Muhamad Amir W Ahmad, Syed Sarosh Mahdi, Zeeshan Sheikh, Umer Daood

**Affiliations:** 1 School of Dental Sciences, Health Campus, Universiti Sains Malaysia, Kubang Kerian, Kota Bharu 16150, Malaysia; 2 Restorative Dentistry Division, School of Dentistry, International Medical University Kuala Lumpur, 126, Jalan Jalil Perkasa 19, Wilayah Persekutuan Kuala Lumpur, Bukit Jalil, Kuala Lumpur 57000, Malaysia; 3 Division of Clinical Oral Health Sciences, School of Dentistry, International Medical University, Kuala Lumpur 57000, Malaysia; 4 Faculty of Dentistry, Dalhousie University, 5981 University Ave, Halifax, NS B3H 1W2, Canada

**Keywords:** riboflavin, crosslinking, collagen, bond strength, adhesive, invitro

## Abstract

The aim of this study was to evaluate published data regarding riboflavin (RF) as a cross-linker for improved adhesive bond strength to dentin and to analyze previous studies for optimal concentration of riboflavin range suitable for dentin bond. Saliva and distilled water were used as storage media and aging time was 24 h and 6 months. Results of meta-analysis were synthesized using a statistical method of inverse variance in random effects with a 95% Confidence Interval (CI). Cochrane review manager 5.4.1 was used to determine results of the meta-analysis. In total, 3172 articles were found from search databases “PubMed”, “Scopus”, and “Google Scholar”. Six of the fifteen studies were eligible for meta-analysis. Micro tensile strength shows significant improvement with the addition of riboflavin (*p* < 0.05) compared to without the addition of riboflavin from with 95% CI. A significant difference has been found in micro tensile bond strength between use of the riboflavin cross-linker and without use of the riboflavin crosslinker in the dentin adhesive system. With a 95% confidence interval (CI), the I^2^ for micro tensile strength was 89% with strong heterogeneity, Chi^2^ = 44.76, df = 5 (*p* < 0.00001), and overall effect size is Z = 2.22 (*p* = 0.03) after immediate aging. Chiang et al. 2013 shows maximum mean differences which is 38.50 [17.93–59.07]. After 6 months of aging in distilled water or artificial saliva micro tensile bond strength has been increased with the addition of riboflavin (*p* < 0.05). It can be clearly seen that pooled effect and 95% CI did not cross the line of no effect. With a 95% confidence interval (CI), the I^2^ for micro tensile strength was 96% with strong heterogeneity, Chi^2^ = 117.56, df = 5 (*p* < 0.00001), and overall effect size is Z = 2.30 (*p* = 0.02). Subgroup analysis proved a similar effect of distilled water and artificial saliva as storage media on micro tensile bond strength after incorporating riboflavin as a collagen crosslinker. An artificial saliva aged forest plot also showed considerable heterogeneity with I^2^ = 96%; Tau^2^ = 257.32; Chi^2^ = 94.37; df = 2 (*p* < 0.00001); test for overall effect, Z = 1.06 (*p* = 0.29). Riboflavin prior to or with bonding is recommended to improve the bonding of different adhesive systems.

## 1. Introduction

The bonding of resin to dentin is an essential strategy for minimally invasive restorative dentistry. This adhesive strategy treats dental defects caused by caries, coronal fractures, and genetic diseases effectively (e.g., amelogenesis imperfecta) [1,2,3]. The durability of resin–dentin bonds is critical for restoration survival and continues to be a clinical challenge [4]. Dentin bonding produces a hybrid layer by demineralizing mineralized dentin and allows adhesive resin monomers to infiltrate into the hybrid layer zone of demineralized dentin, where the monomers polymerize and interlock with the dentin matrix. [5,6]. The difference in depth between acid etching and adhesive penetration, on the other hand, results in the presence of incomplete resin penetration, and denuded collagen fibrils within the hybrid layer. Those exposed collagen fibrils are vulnerable to hydrolysis and enzymatic degradation [7], resulting in further bond instability [8,9]. Furthermore, the resin–dentin interface degrades over time due to dentin collagen degradation mediated by endogenous proteases (e.g., cysteine cathepsins and matrix metalloproteinases (MMPs)); this issue is more visible in the interface created with etch and rinse bonding systems, as the acid-etched dentin matrix lacks proper resin infiltration within the hybrid layer [10,11].

The adhesive–dentin bond is still a multifactorial issue and needs to be studied in detail. However, the incompatibility of hydrophobic photo initiators and hydrophilic monomer composition may compromise initial adhesive polymerization and bonding efficiency, along with factors of the remaining water in interfibrillar spaces of the collagen network, remaining solvent in the adhesive, and phase separation of hydrophilic/hydrophobic monomers within the hybrid layer [12]. No resin–dentin bonding protocol developed to date has been capable of producing perfectly hybridized dentin interfaces in a clinically reasonable time [13,14]. Furthermore, the activation of endogenous enzymes during bonding contributes to the rapid hydrolysis of collagen fibrils [15]. Since collagen fibrils act as reinforcement, the formation of cyclic cracks at resin–dentin interfaces, as well as collagen degradation, invariably results in detrimental weak links. Preservation of the demineralized collagen network over time, protected by a well-formed polymer network, is critical for the durability of hybrid layers [16].

Collagen crosslinking was introduced as an alternative dentin pre-treatment to “reinforce” dentin collagen and improve bonding durability [17,18]. The effects of collagen cross-linkers are attributed to various mechanisms, including maintaining the collagen network in an expanded state, which facilitates inter-diffusion of solvent and hydrophilic monomers, increasing the stiffness of demineralized dentin, with reduction of the plasticization effect caused by water sorption. Moreover, cross-linkers inhibit collagenase activity with a subsequent decrease in the biodegradation rate of collagen within the resin–dentin interface [19]. Riboflavin (RF) in combination with ultraviolet-A (UVA) irradiation has been used in ophthalmology for many years to strengthen the cornea [20]. UVA light high energy (365 nm) breaks down weak intrinsic cross-links in collagen and generates free oxygen radicals. RF attaches to the functional hydroxyl groups including proline or lysine [21]. Furthermore, RF acts as a UVA photosensitizer, promoting the formation of new cross-linkages [22]. Cova et al. [23] reported using RF/UVA inside dentin adhesive treatment. A stable collagen network is required for the formation of a hybrid layer and the prevention of subsequent degradation, and photoactivated RF-induced collagen cross-linking appears to be a promising adjunctive treatment for dentin bonding [23]. However, the data on the best protocol for RF treatments in related applications is insufficient. Recent studies on RF collagen crosslinking and improved dentin hybridization by enhanced adhesive penetration have been performed [24,25,26]. Nevertheless, the desired outcome in terms of bond strength and bond durability remains inconsistent [14]. To the best of our knowledge, no meta-analysis of the effects of RF on dentin bonding properties and bonding efficiency has been published. This systematic review and meta-analysis will aim to evaluate the published data regarding RF concentration as a cross-linker associated with the medium of aging for better bond strength of dentin. In addition, previous studies will be evaluated for the optimum concentration of riboflavin suitable for dentin bonds; moreover, the literature will be compared for the micro tensile bond strength after riboflavin-induced adhesive treatment. The objective of our review was to evaluate the modification of micro tensile bond strength after incorporating photo-activated riboflavin in a dentin bonding/adhesive system.

## 2. Materials and Methods

### 2.1. Study Design

For this systematic review, PRISMA guidelines for reporting systematic reviews have been followed and the protocol was registered in PROSPERO on 20 December 2021 (registration number: CRD42022382903) and last edited on 5 January 2022.

The present systematic review of in vitro studies is conducted according to Preferred Reporting Items for Systematic Reviews and Meta-Analyses [27] (PRISMA) guidelines PICO.

- P = Sectioned dentin from extracted human teeth
- I = Teeth modified with UVA/Blue light/photoactivated Riboflavin as a crosslinker for the dentinal bond.
- C = Teeth modified without the use of Riboflavin crosslinker
- O = Micro tensile bond strength

### 2.2. Eligibility Criteria

Inclusion criteria:

All in vitro studies associated with photoactivated “riboflavin” as a crosslinking agent applied for the dentin bond were included. In addition, only those articles that compared dentin bond strength on human extracted teeth were considered. 

Exclusion criteria:

Articles that discussed bond strength without associating riboflavin and did not test the micro tensile bond of dentin were excluded. In addition, articles in other languages other than English, simple cross-sectional studies, cohort studies, clinical trials, review articles, and case reports were not made part of the review. Any disagreements on study inclusion and exclusion criteria were discussed and resolved by consulting a third reviewer.

### 2.3. Research Question

Based on the Preferred Reporting Items for Systematic Review and Meta-Analysis (PRISMA) guidelines, a specific question was constructed. The addressed focused question was “Does incorporation of riboflavin as a crosslinker improve the micro tensile bond strength of dentin bond?”

### 2.4. Information Sources

There were two independent reviewers who screened the titles and abstracts of all the identified studies to determine the relevance meeting the pre-determined inclusion criteria. The authors searched the “PubMed”, “Scopus”, and “Google Scholar” databases from January 1970 up to and including December 2022 for appropriate articles addressing the focused question (Table 1). The search for the review started on 27 December 2021 and ended on 29 December 2022. The full text was considered if there were sufficient data to make clear decisions. A structured and logical approach to the literature search was used to identify the relevant papers that report collagen crosslinking, bonding efficacy, and RF. Reference lists of original studies were hand-searched to identify any articles that could have been missed during the initial search, keeping the inclusion criteria in mind. 

### 2.5. Search and Study Selection

A table showing the Search strategy in PubMed, Scopus, and Google Scholar is provided below.

### 2.6. Data Collection

The search was done according to the previously mentioned search strategy from three databases. After obtaining all search outcomes based on title screening, duplicates were removed by using Endnote X8.2. and then abstract screening was done followed by a full paper read. Articles that were selected for full-paper reading were reviewed by both reviewers. Any disagreements regarding study selection were resolved via discussion.

### 2.7. Search Item (Data Extraction)

The developed data extraction was used to extract information from the included studies. Data extraction from the selected studies consists of the name of the author, year, crosslinking agent, percentage of crosslinking agent, aging condition, adhesive system used, and micro tensile strength property. The measurement of the micro tensile strength property is summarized by MPa.

### 2.8. Meta-Analysis Criteria

Articles were selected for meta-analysis based on the following criteria:

(i) All vitro studies used 0.1% photoactivated riboflavin as a collagen crosslinker which is considered a case group.
(ii) Studies used a control group without the presence of riboflavin.
(iii) Micro tensile strength has been reported during the evaluation of dentin bonding.
(iv) Micro tensile strength has been compared between the case and control group
(v) Sample size and mean (SD) of microtensile bond strength has been reported for both case and control group
(vi) Micro tensile bond strength has been assessed after both immediate (24 h) and long-term (6 months) storage duration
(vii) Distilled water or saliva has been used as storage media.

## 3. Reporting Biases

Using a six-item quality rating form developed by the Cochrane Collaboration, two writers evaluated the methodological quality of the studies independently. This tool assesses the following factors: the randomized process, the concealment of allocation, blinding, the extent of follow-up, the publication of only some results, and additional sources of bias [28]. We reported any significant concerns regarding bias, such as any baseline imbalance in factors closely associated with outcome measures, that were not addressed in the other domains in accordance with the QUIN Tool [29]. A team of doctors and researchers created this method to address the difficulties encountered while assessing the quality of vitro studies. Additionally, this application offers a technique to calculate the bias risk of in vitro experiments, enabling users to compare the caliber of various investigations. Clinicians and researchers involved in developing this tool finally set the score for each of the 12 criteria as adequately specified = 2 points, inadequately specified = 1 point, not specified = 0 point, and not applicable = exclude criteria from the calculation. The scores were then combined to obtain a final score for a specific in vitro experiment. The in vitro study was rated as high, medium, or low risk using the scores that were achieved (>70% = low risk of bias, 50 to 70% = medium risk of bias, and <50% = high risk of bias) by using the formula: Final score = (Total score × 100) /(2 × number of criteria applicable).

Two authors (NBJ and UMD) separately input information regarding the study’s methodologies, participants, interventions, and outcomes into a data extraction form. Any discrepancies were settled by discussion and collaboration with a third author, as well as by referring to the trial report (SUM).

### Synthesis of Results

The results of the meta-analysis were synthesized using a statistical method of inverse variance in random effects with a 95% Confidence Interval (CI). Cochrane review manager 5.4.1 was used to determine the results of a meta-analysis. Cochrane review manager was used to obtain statistical heterogenicity *p* values, z values, and subsequent subgroup analysis with a forest plot.

## 4. Results

### 4.1. Study Selection

A multi-step search approach was applied to retrieve pertinent literature from the three electronic databases “PubMed”, “Scopus”, and “Google Scholar”, a total of 3172 articles were found from the search databases. A predetermined search strategy was used for searching the articles. A search strategy was developed on PubMed and adapted for the other databases. The search strategy utilized the listed key terms for subject headings: “riboflavin” AND “tooth” OR “teeth” OR “enamel” OR “dentin” OR “collagen” OR “dentin-bonded” OR “dentin bonding”. Also “riboflavin” AND OR “enamel” OR “dentin AND “micro tensile bond strength” was used as the search strategy. The search was performed by Boolean operators (“AND”, “OR”, and “NOT”) or subject heading truncations (*), depending on the necessity of relevant databases. Two independent authors (SZE and AB) identified literature regarding the research question, then had run a screening using titles, and reviewed abstracts throughout the literature search. The third author (NBJ) was also involved in the study selection process for this review in order to resolve discrepancies and biases. After that, the final selection of the literature was performed by two authors (SZE and AB). They reviewed full texts and matched them with eligibility criteria to include studies according to research objectives. Database searches were re-run by author UMD before the final synthesis and performed analysis to avoid the exclusion of the latest relevant. The search was limited to literature found in the English language only because we were not financially eligible or did not have enough logistic support to retrieve and translate articles published in other languages except English.

**Figure 1:**
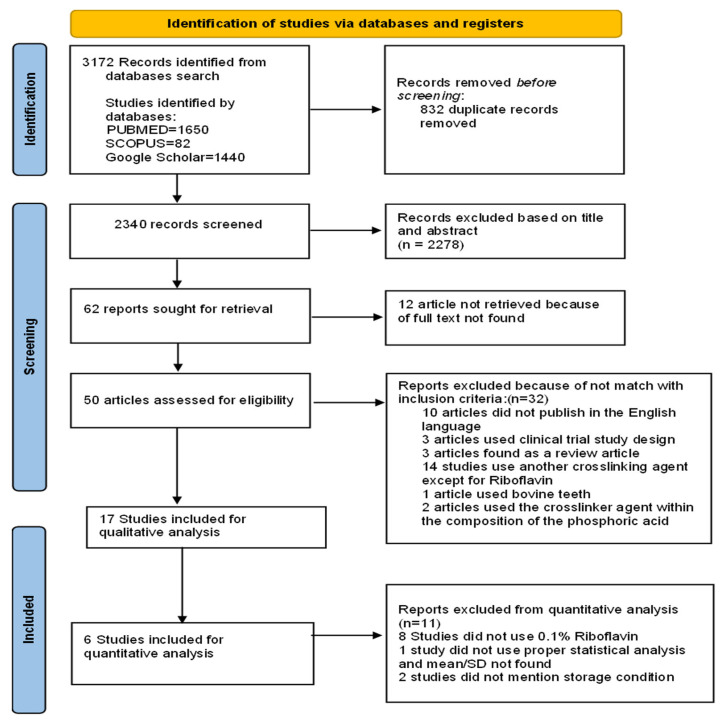
PRISMA flow chart of the study. PRISMA 2020 flow diagram for new systematic reviews which included searches of databases and registers only.

The PRISMA flow chart (Figure 1) illustrates an overview of the article screening process, from the initial search to the complete paper review. The full paper review resulted in 15 papers being selected.

From all three databases (PubMed, Scopus, Google Scholar), a total of 3172 papers were retrieved. A total of 2340 documents were reviewed after the duplicates had been removed by reading the title and abstract. A total of 2278 articles were subsequently eliminated because they failed to meet the criteria for inclusion, leaving 62 items in total for full-text reading evaluation. Of these articles, for 12 studies, full texts could not be retrieved. In total, 50 articles were assessed for eligibility, but among them, 33 were not considered in the qualitative analysis because, among the 10 studies published in different languages except for the English language, 3 studies used clinical trial study design, 3 studies were found as a review article, 14 studies used another crosslinking agent except for riboflavin, 1 article used bovine teeth, and 2 articles used the crosslinker agent within the composition of the phosphoric acid.

A total of 17 articles were included in the qualitative analysis. Of these, 11 articles were not considered for the meta-analysis. The reason is that of these 11 studies, 8 did not use 0.1% riboflavin, 1 did not use proper statistical analysis and mean/SD was not found, and 2 of these did not mention storage conditions.

A flowchart designating the study selection process according to the PRISMA statement is presented in Figure 1.

### 4.2. Synthesis of Meta-Analysis

Meta-analysis was conducted with six studies. The meta-analysis evaluated the micro tensile bond strength of the dentin bonding system with and without the use of riboflavin [18,23,26,30,31,32] because these studies exhibit similar findings.

The results were presented using a random-effects model, and the pooled estimate of 0.1% photoactivated riboflavin was obtained with a 95% confidence interval (CI). The meta-random-effects analysis’s approach was thought to be more suitable for the current investigation. Mean, SD of microtensile bond strength with or without application of RF, and sample size of the study were retrieved and used for meta-analysis. When there is a significant amount of heterogeneity among the included research, the random-effects model weighs the studies more consistently and is thought to be more appropriate. To determine the correlation between study and estimate heterogeneity, Cochran’s Q test (χ^2^) and the I^2^ statistic were used in the form of percentages. *p* value < 0.05 will be considered as statistically significant. High heterogeneity in estimates from different research, as shown by a larger percentage from the I^2^ statistics where (I^2^ < 20 % reveals low heterogeneity; 30–70% shows moderate heterogeneity and >75% exhibits high heterogeneity). A forest plot was used to present the combined results of the study to explore effect of microtensile bond strength changes by using photoactivated riboflavin in dentin adhesive system with a 95% confidence interval (CI). The analysis was conducted by using Revman, Cochrane’s Review Manager 5.4.1 software, USA.

**Figure 2:**
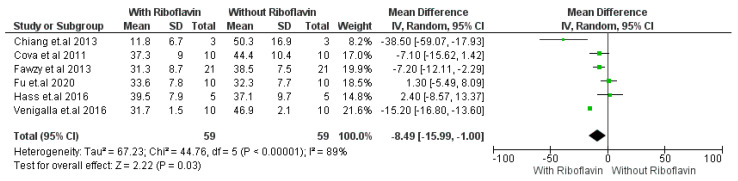
Forest plot of micro tensile bond strength of adhesive with 0.1% RF crosslinker-i. Metaanalysis evaluated the micro tensile bond strength of the dentin bonding system with and without the use of 0.1% riboflavin [18,23,26,30,31,32] after immediate (24 h) storage.

In Figure 2, micro tensile bond strength without the use of 0.1% riboflavin was considered as control group and micro tensile strength with the use of riboflavin was used as study group. Micro tensile strength shows significant improvement with the addition of riboflavin (*p* < 0.05) compared to without the addition of riboflavin from with 95% CI. A significant difference has been found in micro tensile bond strength between use of the RF crosslinker and without use of the RF crosslinker in the dentin adhesive system. With a 95% confidence interval (CI), the I^2^ for micro tensile strength was 89% with strong heterogeneity, Chi^2^ = 44.76, df = 5 (*p* < 0.00001), and overall effect size is Z = 2.22 (*p* = 0.03). Chiang et al. 2013 shows maximum mean differences which is 38.50 [17.93–59.07] in micro tensile bond strength among all the studies after adding RF crosslinker in the dentin–resin bonding after 24 h aging. However, Fu et al. 2020 [26] and Hass et al. 2016 [31] showed negative changes in in micro tensile bond strength after adding RF crosslinker in the dentin–resin bonding. Except these two studies, other studies (Chiang et al. 2013, Cova et al. 2011, Fawzy et al. 2013, and Venigalla et al. 2016) [18,23,30,32] improvement in microtensile bond strength after incorporating RF collagen crosslinker in the adhesive system. Pooled effect of six studies showed positive increase in microtensile bond strength after RF use.

**Figure 3:**
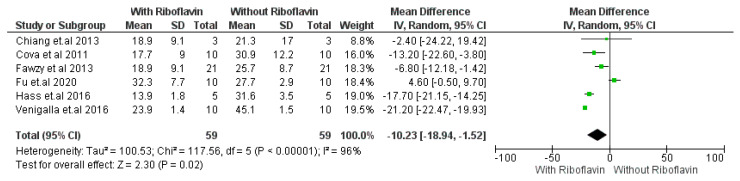
Forest plot of micro tensile strength property of adhesive with 0.1% RF crosslinker. Metaanalysis was conducted with six studies. The meta-analysis evaluated the micro tensile bond strength of the dentin bonding system with and without the use of riboflavin [18,23,26,30,31,32] after prolonged (6 months) storage.

In Figure 3, after a storage duration of 6 months in artificial saliva or distilled water, micro tensile bond strength was evaluated with or without adding 0.1% RF as collagen crosslinker. Adhesive without the use of riboflavin crosslinker was considered as the control group, and adhesive with the use of riboflavin was considered as the study group. From the analysis, significant improvement of microtensile bond strength has been shown with the addition of riboflavin (*p* < 0.05) compared to without the addition of riboflavin from included studies. It can be clearly seen that pooled effect and 95% CI did not cross the line of no effect. With a 95% confidence interval (CI), the I^2^ for micro tensile strength was 96% with strong heterogeneity, Chi^2^ = 117.56, df = 5 (*p* < 0.00001), and overall effect size is Z = 2.30 (*p* = 0.02). From the result, we can observe that Venigalla et al. 2016 exhibited maximum changes in microtensile bond strength while Chiang et al. 2013 [30] showed the minimum alteration after incorporating RF collagen crosslinker in the dentin-resin bonding. However, only Fu et al. 2020 showed negative modification in microtensile bond strength after incorporating RF collagen crosslinker. Pooled effect shows improvement of microtensile bond strength after using RF collagen crosslinker with 6 months of storage.

### 4.3. Subgroup Analysis

**Figure 4:**
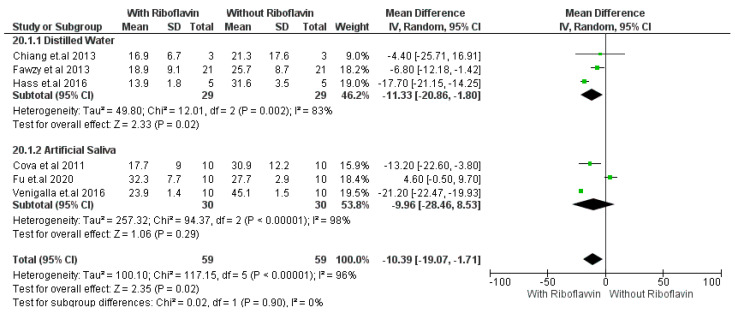
Subgroup analysis of micro tensile strength property of adhesive using 0.1% RF-crosslinker [18,23,26,30,33,34].

The subgroup analysis was performed to explore the aging effect on the micro tensile bond strength of 0.1% RF-induced dentin bonding. The comparison was performed between distilled water and artificial saliva aged micro tensile bond strength of 0.1% RF-induced dentin bonding. (Figure 4). Distilled water-aged dentinal bonds showed considerable heterogeneity because I^2^ was found to be 83%. Additionally, Tau^2^ = 49.8; Chi^2^ = 12.01; df = 2 (*p* = 0.02); test for overall effect, Z = 2.33 (*p* = 0.02).

Artificial saliva-aged forest plot also showed considerable heterogeneity with I^2^ = 96%; Tau^2^ = 257.32; Chi^2^ = 94.37; df = 2 (*p* < 0.00001); test for overall effect, Z = 1.06 (*p* = 0.29). Further analysis of the subgroup revealed that for both distilled water and artificial saliva, the pooled effect and 95% CI did not cross the line of no effect, which means both the storage media distilled water and artificial saliva influence improvement of micro tensile bond strength changes in a positive way in the case of 0.1 % RF-induced dentin bonding after 6 months of storage.

The overall quality of evidence in the selected studies was classified as low, with the majority of the studies achieving a low risk of bias.

## 5. Discussion

The current systematic review investigated effect of RF collagen crosslinker in modifying microtensile bond strength of resin dentin adhesive. Overall, all studies included indicated that the incorporation of riboflavin to adhesive improved the bond strength. There was an extensive search and rigorous screening performed of the literature to reduce heterogeneity within the meta-analysis. However, the data sets obtained still showed some level of heterogeneity, which may have been due to the different types of bonding systems. Subgroup analyses were conducted for this reason exploring some new formulations that have been defined and modified from previous protocols of corneal collagen [35] and dentin collagen [36] crosslinking. Riboflavin forms a defensive shield against UVA absorption and produces a bond between collagen crosslinking and the proteoglycan-core proteins [37]. The presence of RF is known to reduce the degradation of the resin–dentin interface via the bonding of proline or lysine to the available functional hydroxyl groups inside the collagen network [38]. Being a crosslinking agent, Rf can stiffen the collagen fibrils [39] and interfere with the mobility of proteases [40] and maintaining telopeptides as a result of blockage of collagenases and inactivation of C-terminal telopeptides, eventually binding to critical peptide bonds [41]. The blue light used in different studies might have a dual effect in activating RF and simultaneously activating the polymerization reaction of the resin monomers of the experimental adhesives used, thus maintaining the resin dentin bond.

The use of riboflavin as a photoinitiator in dentin adhesive resulted in improved bond strength and better antimicrobial capability with excellent degree of conversion [42]. Our study results also show improvement of microtensile bond strength after adding the RF crosslinker. A study by Fawzy et al. (Table 2 and Table 3) was a proof-of-concept strategy for avoiding the problem of collagen degradation with RF modified inside two-step etch-and-rinse dentin adhesives minimizing degradation of the adhesive interface after 3 years of storage [29]. In another study, resin–dentin micro-tensile bond strength had been found to be reduced after 6 months of aging after incorporation of 0.1% RF with 1 week of aging modified inside Zip bond. Blue light photoactivated 0.1% riboflavin-modified adhesives improved the biochemical and biomechanical properties of demineralized dentin as well as the long-term resin–dentin interfacial integrity and bond strength of universal adhesives to dentin [26]. In addition, the incorporation of RF with quaternary ammonium antimicrobials at a concentration of 1% within the adhesive may provide a long-term antimicrobial effect and simultaneously cross-linking dentin matrix for clinicians improving the immediate bond strength and bond durability without adversely affecting the degree of conversion in the adhesive monomers [39].

Table 4 presented low risk of bias for the six studies included in the meta-analysis.

The micro tensile bond strength of dentin adhesive has shown significant improvement with the addition of riboflavin from the varying results (Figure 2). Recent research has shown a physical process of crosslinking which has been known as photo-oxidative homologous recombination. This method employs riboflavin, a popular crosslinker. Riboflavin develops an excited or triplet state that releases reactive oxygen radicals when it absorbs ultraviolet light A (UVA light), resulting in effective physical crosslinking of a collagen network. The ability of UVA-activated riboflavin to improve the mechanical characteristics of both demineralized and non-demineralized dentin has been demonstrated [47]. Riboflavin boosted initial bonding strength to dentin and lowered interfacial nano leakage and MMP activity in aged specimens when used in an experimental primer to crosslink dentinal collagen [48]. According to Cova et al. [31], the ability to cross-linking compounds to make dentin collagen more rigid has a stabilizing effect on dentin matrix decomposition. This work provides significant evidence that pre-treatment with riboflavin and UVA can also neutralize MMPs, particularly MMP-9. Mineralized dentin contains latent MMPs that can be reactivated in low-pH situations [49]. After eliminating the mineral component from dentin, an etch-and-rinse adhesive is applied, exposing endogenous MMPs and inducing an activation process coupled to the acidity of the etchant and adhesive [50]. We hypothesize that UVA-activated riboflavin may limit MMPs activity through effectively cross-linking MMPs as well as by fortifying the collagen fibrils through cross-linking. Collagenase activity against insoluble bone collagen requires telopeptidase activity of osteoclast-derived MMP-9, which separates collagen molecule telopeptides [51]. Due to decreased MMP-9 telopeptidase activity, riboflavin-induced MMP-9 blockage may be accountable for the hybrid layers’ enhanced persistence. When exposed to ultraviolet-A (UVA) radiation, RF has the capacity to generate radicals (^1^O_2_), and collagen crosslinking is accomplished by photo-oxidation, which forms covalent intermolecular crosslinks. In addition, it was recommended that riboflavin/UVA be added to dentine adhesives to strengthen the bond immediately, maintain the adhesive interface, inhibit dentine matrix metalloproteinases, and lengthen the longevity of the resin-dentin bond [36].

Our study indicated that Fu et al. [26], Cova et al. [23], and Venigalla et al. [32] were using artificial saliva as storage media whereas Hass et al. [43], Chiang et al. [42], and Fawzy et al. [18] were using distilled water. The subgroup analysis revealed that the ageing with artificial saliva or distilled water influences micro tensile bond strength changes in a similar way in the adhesive system after incorporating RF crosslinker (Figure 3). Storage solutions had a considerable impact on the mineral concentrations of Ca, K, Na, and P present in dentin, and tooth samples preparing for mechanical testing often undergo an aging process to preserve hydration and mineral concentration of dentin [52]. Since the compositional structure of dentin was changed by the storage solution and time, it is essential to identify and employ the proper storage method to maintain the properties of micro elastic tissue [53]. Dentin is composed of 70% minerals, 20% organic materials, and 10% water, while the content of enamel is as follows: 95% mineral content, 4% organics, and 1% water. The impact of a storage solution on dentin should be much more dissimilar from that on enamel as a consequence of structural composition [52]. Raum et al. found in their vitro study that artificial saliva as a storage media did not influence the elastic properties of dentin whereas saline solution causes 70% reduction in the acoustic impedance in dentin [51]. Saline can dissolve minerals from dentin rapidly when it is stored for the required time. However, in recent years, saline is considered the most popular storage media due to its availability and cheaper price. Study results proved that Storage procedures can alter the proportion of mineral contents of dentin (Ca, K, Na, and P) [52,54]. Eltoukhy et al. described strong influence of different aging media and aging time on the microtensile bond strength but our study results found a similar effect of storage media and aging time (24 h and 6 months) in positive alteration of micro tensile bond strength [55].

The micro tensile bond strength of different dentin adhesive systems (Adper Scotchbond Multi-Purpose; SBMP), (Clearfil SE Bond, CSE), (Clearfil S3 Bond, S) amongst different studies revealed no significant difference after 24 h of aging in both artificial saliva and distilled water; however, after thermocycling, all tested adhesive systems showed significant difference [53].

In vitro bond strength studies have multiple variables such as specimen base area, the concentration of modifier, composite, curing light, and techniques to place the composite which could have affected the results. Due to the overall limited number of studies, we did not consider the adhesive strategy or the kind of adhesive when analyzing the different surface pretreatments. However, potential interactions between the kind of surface pretreatment and the applied adhesive might affect the results. These have not been factored into this review. Adhesion promoters have also been excluded from the study. The obtained results should be interpreted with caution because the analysis indicated a high level of heterogeneity and sufficient evidence to support a clinical decision is unavailable. Riboflavin crosslinking is the future direction of dental adhesives and studying the available materials and identifying problems that can help improve resin–dentin bonds are important endeavors. Randomized clinical studies with long-term follow-ups are recommended to obtain strong clinical evidence that can help clinicians in their decision-making process.

## 6. Conclusions

A significant difference has been found between riboflavin-induced crosslinker and without riboflavin-induced crosslinker in terms of improving the micro tensile bond strength of dentin. Use of distilled water or artificial saliva as storage media showed a similar effect in increasing of micro tensile bond strength in dentin bonding after incorporating a photo-activated RF-based crosslinker. In addition, after 24 h of aging and 6 months of aging shows improvement in micro tensile bond strength with the use of RF collagen crosslinker compared to without use of RF.

## Figures and Tables

**Table 1:** Search strategy.

Date of Searching	Database	Search Strategy
28 December 2021	PubMed	((((((Riboflavin [Title/Abstract]) AND (Dentin [MeSH Terms])) AND (dentin bonding agents [MeSH Terms])) OR (dentin bonding [MeSH Terms])) OR (collagen crosslinker [Title/Abstract])) OR (collagen crosslinking agent [Title/Abstract])) OR (microtensile bond strength [Title/Abstract])
27 December 2021	Scopus	“(TITLE-ABS-KEY (“Riboflavin”), TITLE-ABS-KEY (“Dentin”), AND TITLE-ABS-KEY (“Bonding”) OR TITLE-ABS-KEY (“micro tensile bond strength”) OR TITLE-ABS-KEY (“Shear bond”) OR TITLE-ABS-KEY (“dental adhesion”) OR TITLE-ABS-KEY (“Adhesive”) OR TITLE-ABS-KEY (“bonding agent”)) OR TITLE-ABS-KEY (“Collagen crosslinking agent”) OR TITLE-ABS-KEY (“Collagen crosslinker”) AND (LIMIT-TO (LANGUAGE, “English”))”
29 December 2021	Google Scholar	Riboflavin AND (Dentin) AND (dental adhesive) OR (Dental bonding) OR (dental bonding agent) OR (Dental adhesion) OR (Collagen crosslinker) OR (collagen cross-linking agent) OR (Shear bond strength) OR (micro tensile bond strength)

**Table 2:** Data extraction from included studies.

Author (Year)	Crosslinker	Storage Condition	Adhesive System	Adhesive Strategy	Bond Strength Measured by-	Author’s Remark
Cova et al. (2011)	UVA activated riboflavin (0.1%)	Artificial saliva for 24 h, 6 months, or 1 year	XP Bond adhesive (Dentsply)	Pretreatment	µ-TBS	Role of cross-linking agents on dentinal MMPs was not explained in this paper [23]
Fawzy et al. (2012)	Blue light activated riboflavin/UVA (0.1% and 1%)	Distilled water at 37 °C for 4 months	Cyanoacrylate adhesive (Zapit, Dental Ventures of America, Corona, CA, USA)	Pretreatment	µ-TBS	visible blue light showed to be a promising substitute for UVA as it is clinically more applicable and acceptable [17]
Fawzy et al. (2013)	Blue light or UVA activated riboflavin (0.1%)	Distilled water at 37 °C for 24 h and 6 months	Cyanoacrylate adhesive (Zapit, Dental Ventures of America, Corona, CA, USA)	Pretreatment	µ-TBS	In all modified and control groups, there was a significant drop in µTBS after 6 months of aging in distilled water when compared to baseline measures taken at 24 h [18]
Chiang et al. (2013)	UVA activated riboflavin (0.1%, 1%)	Distilled water at 37 °C 24 h and 6 months	Scotchbond Multi-purpose primer and adhesive (3 M/ESPE, St. Paul, MN, USA)	Pretreatment	µ-TBS	Application of 0.1 percent RF under 2-min UVA treatment was effective for improving resin-dentin bonding [30]
Daood et al. (2014)	Blue light activated riboflavin (1, 3, 5, 10%)	Artificial saliva for 24 h and 9 months	The two-step Adper^TM^ Single Bond adhesive	The experimental adhesive was prepared by incorporating bis-GMA, HEMA and visible light photo-initiators into ethanol. Adhesive-system was then modified with 0, 1.0, 3.0, 5.0% and 10.0% of Riboflavin.	µ-TBS	The addition of RF to the experimental two-step etch-and-rinse adhesive at a concentration of 3% enhanced bond strength and durability after 9 months in artificial saliva storage without altering the degree of conversion in the adhesive monomers [43]
Mohamed Hashem (2021)	UVA activated Riboflavin (0.1, 0.5%)	Distilled water (37 °C) Duration: Immediate and One month	Zapit adhesive (Dental Ventures of America, Corona, CA, USA)	The 0.1 and 0.5% riboflavin solutions were prepared separately and solutions were then mixed in a commercially available dentin adhesive system.	µ-TBS	Riboflavin concentration has a significant impact on resin–dentin micro tensile bond strength [42]
Daood et al. (2018)	UVA activated Chitosan/ riboflavin (Undefined)	i. 24 h in distilled water (37 °C) ii. 48 h at artificial saliva	cyanoacrylate adhesive (Zapit, Dental Ventures of North America, Corona, CA, USA).	Chitosan/ riboflavin (Ch/RF) solutions were prepared in four ratios of 1:1, 1:2, 1:3, and 1:4 by volume. The pH of formulated solutions was kept to levels of 6.0 and 1.0 N. After that, formulations were mixed with ethanol-based monomers and acetone-based monomers. To obtain a well-mixed RF/solvent/monomer solution, shaking and sonication processes were applied.	µ-TBS	The concentration of cross-linkers was not mentioned in this paper. Aging time significantly affects resin–dentin micro tensile bond strength [33]
Fu et al. (2020)	Blue light activated riboflavin (0.1%)	Artificial saliva. 1-week and 6-month storage	UA Scotch bond	The experimental adhesive was prepared using hydrophobic monomer (decandiol methacrylate/D3 MA) bis-GMA, and HEMA (2-hydroxyethyl methacrylate) at a mass ratio of 10:60:30. Ethanol was added as a solvent at a concentration of 30% (m/m). Camphorquinone (0.5%, m/m) DPIHP (1.0%, m/m) and ethyl (4- dimethylamino) benzoate (0.5%, m/m) were used as photoinitiator. Methacryloyloxydecyl Dihydrogen phosphate/14% (MDP) is added as a mild hydrophilic monomer. At a sub-minimal ratio of 5%, n, biphenyl dimethacrylate (5%) (BPDM), dipentaerythritol pentacrylate phosphoric acid ester (PENTA) were added. This experimental adhesive was then modified by adding 0.1% RF.	µ-TBS	Resin–dentin micro-tensile bond strength has been found to be reduced after 6 months of aging [26]
Hass et al. (2016)	UVA-activated. Riboflavin (0.1%), ii. Proanthocyanidin iii. Glutaraldehyde	Distilled water 24 h 18 months	Single Bond Plus (SB) (3 M ESPE, St. Paul, MN, USA) Batch number N531785	Pretreatment	µ-TBS	Glutaraldehyde substantially decreased cell viability and it is recommended to avoid in clinical settings [31]
Venigalla et al. (2016)	i. UVA activated riboflavin (0.1%) ii.1-Ethyl-3-(3-dimethylaminopropyl) carbodiimide (EDC), iii. Proanthocyanidin, a natural cross-linker	Artificial saliva for 24 h and the other after 6 months	Adper Single Bond Adhesive	Pretreatment	µ-TBS	When compared to other chemical cross-linking agents, UVA–riboflavin photo-oxidative cross-linking was more successful in improving resin–dentin binding strength and durability [32]
Hass et al. (2016)	UVA activated riboflavin (0.1%)	No aging	Scotchbond etchant (3 M ESPE) Single Bond Plus (3 M ESPE, St).	Pretreatment	µ-TBS	Although glutaraldehyde proved efficient in stabilizing resin–dentin contacts, its clinical application is controversial due to the risk of cytotoxicity [44]
Abunawareg et al. (2017)	Blue light or UVA activated Riboflavin (1%)	Distilled water at 37 °C for 24 h, 6 months, or 12 months	cyanoacrylate adhesive (Zapit, Dental Ventures of America Inc., Corona, CA, USA).	Pretreatment	µ-TBS	Dental collagen cross-linking produced by UV or blue light-activated 1 percent riboflavin or EDC-HCl improved the resin–dentin bond’s endurance and strength [45]
Gajjela et al. (2017)	UVA activated 1% riboflavin	No aging	cyanoacrylate adhesive	Pretreatment	µ-TBS	When the dentin surface was prepared with riboflavin/chitosan, as opposed to the control group, an increase in the bond strength of resin composite to dentin was obtained [34]
Abuelenain et.al (2018)	Blue light or UVA activated riboflavin (1%)	Distilled water at 37 °C for 24 h	(Scotchbond^TM^ Universal Etchant, 3 M ESPE)	Pretreatment	µ-TBS	Acid etching of dentin reduced hardness, and the use of proposed cross-linking substances did not improve the hardness or μTBS [46]
Daood et al. (2020)	UVA activated riboflavin (0.125%)	Artificial saliva for 24 h and 12 months	experimental adhesive system based on bis-GMA, HEMA and hydrophobic monomer	Bis-GMA, HEMA and hydrophobic monomer was doped with RF0.125 (RF—Riboflavin) or RF/VE-TPGS (0.25/0.50) used as an experimental adhesive.	µ-TBS	After 24-h aging in artificial saliva, the RF/VE-TPGS0.25 groups had the strongest µTBS of dentin bonding [24]
Daood et al. (2020)	UVA activated riboflavin (0.5, 1, 2%)	Distilled water at (37 °C) for 24 h.	cyanoacrylate adhesive (Zapit, Dental Ventures of North America, Corona, CA, USA).	Experimental adhesives modified with different fractions of dioctadecyldimethyl ammonium bromide quaternary ammonium and riboflavin (QARF).	µ-TBS	The use of QARF at 1% in an experimental two-step etch-and-rinse adhesive enhanced immediate bond strength and bond endurance without compromising bond integrity [24]
Daood et al. (2021)	UVA activated riboflavin (0.125%)	Distilled water at 37 °C for 12 months	Adper Scotchbond™ Etchant, Adper™ Single bond 2	Riboflavin-5-phosphate solution was used with mixture of novel k21 PLGA nanoparticles and VE-TPGS in the adhesive system	µ-TBS	No significant information found about resin–dentin micro tensile bond strength [25]

**Table 3:** List of excluded papers from meta-analysis and systematic data extraction screening.

Author (Year)	Reason of Exclusion
Mohamed Hashem (2021) [42]	Inadequate information on mean/SD.
Daood et al. (2018) [33]	The concentration of riboflavin used is undefined in the study.
Daood et al. (2020) [24]	The articles do not mention using 0.1% of riboflavin.
Daood et al. (2021) [25]	Articles evaluated riboflavin concentration rather than 0.1%.
Daood et al. (2019) [24]	Articles mentioned riboflavin concentrations used except for 0.1%.
Fawzy et al. (2012) [17]	No sample size.
Abunawareg et al. (2017) [45]	Articles mentioned the concentration of riboflavin used rather than 0.1%.
Daood et al. (2014) [43]	Articles tested riboflavin in different concentrations but not 0.1%.
Abuelenain et al. (2018) [46]	Articles mentioned riboflavin concentrations used except for 0.1%, no aging had been performed.
Gajjela et al. (2017) [34]	Articles mentioned riboflavin used in concentrations except for 0.1%. Aging was not performed.
Hass et al. (2016) [44]	No information regarding storage had been provided.

**Table 4:** Risk of bias of the included study.

Name of Study	Clearly Stated Aims/Objectives	Detailed Explanation of Sample Size Calculation	Detailed Explanation of Sampling Technique	Details of Comparison Group	Detailed Explanation of Methodology	Operator Details	Randomization	Method of Measurement of Outcome	Outcome Assessment Details	Blinding	Statistical Analysis	Presentation of Results	Total Score	Final Score	Overall Bias
Chiang et al., 2013 [30]	2	1	0	2	2	2	2	2	2	1	2	2	20	83.3	Low
Cova et al., 2011 [23]	2	1	0	2	2	2	2	2	2	1	2	2	20	83.3	Low
Fawzy et al., 2013 [18]	2	1	0	2	2	2	2	2	2	1	2	2	20	83.3	Low
Fu et al., 2020 [26]	2	1	0	2	2	2	1	2	2	1	2	2	19	79.2	Low
Hass et al., 2016 [31]	2	1	0	2	2	2	2	2	2	1	2	2	20	83.3	Low
Venigalla et al., 2016 [32]	2	1	0	2	2	2	1	2	2	1	2	2	19	79.2	Low

## Data Availability

Not applicable.
